# Social policies as determinants of health: new evidence, ongoing challenges, and future pathways

**DOI:** 10.1093/haschl/qxaf193

**Published:** 2025-10-09

**Authors:** Rita Hamad

**Affiliations:** Department of Social and Behavioral Sciences, Harvard School of Public Health, 677 Huntington Avenue, Boston, MA 02115, USA

**Keywords:** social policy, health equity, administrative burden, policy evaluation

## Abstract

Despite acknowledgment that social and economic policies fundamentally shape health, persistent geographic and sociodemographic inequities in the United States reflect the deliberate choices embedded in policy decisions. There is a critical need for policy research that illuminates not just associations of social policies with health, but also mechanisms and pathways to equitable impact. The special collection of articles in *Health Affairs Scholar* on “Intersections of Social Policies and Health” advances the field by examining how the effects of social policies are shaped by legal, political, and cultural contexts; the uneven implementation and enforcement that contribute to health disparities; and the crucial role of narratives and administrative processes in mediating policy impact. Through nuanced analyses—such as exploring policy ecosystems, administrative burdens, and media framing—these studies move beyond single-policy assessments to explore the complex realities of translating policy intent into population health improvements. Collectively, the collection points toward new research priorities: advancing nuanced measurement of policy contexts, integrating equity and subgroup analyses, prioritizing implementation science, incorporating political and narrative determinants, and embracing intersectoral approaches. By deepening our understanding of how and why policy effects unfold unevenly, this scholarship charts a course for more effective and equitable policy research and action.

Despite longstanding recognition that social and economic policies play a pivotal role in shaping health outcomes, the United States remains defined by striking geographic and sociodemographic inequities in both health and the policy environments that influence it.^1^ These disparities are not mere artifacts. They are often consequences of intentional policy decisions—at the federal, state, and local levels—that influence upstream drivers like income, housing, education, nutrition, and other social determinants of health. The COVID-19 pandemic sharpened focus on these divides, exposing the fragility of the social safety net while also providing opportunities to study the effects of policy changes.^2^ These realities underscore the urgency of research that does more than document associations between social factors and health; it must also guide effective, equitable policies and interventions.

The articles gathered in this special collection mark important progress in answering these demands by asking timely and policy-relevant questions. Moving beyond established findings, they delve into which populations benefit from social policies, how those benefits are mediated by political, administrative, and cultural contexts, and what barriers prevent policies from realizing their full potential. Collectively, this scholarship does more than affirm that upstream policies matter; it interrogates how and why their effects unfold unevenly—and what can be done to translate policy goals into population health improvements. Here, I review the major themes that were highlighted by this special collection, and the challenges and future directions for this field.

## Deeper understanding: social policy, inequity, and the importance of context

A dominant theme across this collection is the recognition that the impact of social policy on health is profoundly shaped by context—legal, political, social, and historical. Earlier literature has established that state-level policy environments influence population health, with many studies treating policy as a binary exposure and examining short-term effects at the population level. The papers here advance the conversation in critical ways by demonstrating the nuanced features of policy implementation and effects.

For example, one study in this collection examines the long-term impacts of early education policy—in the form of Head Start—demonstrating impacts later in life (in this case, on measures of interpersonal violence^3^) that are often missing in government cost-benefit analyses. Several studies interrogate not just the existence of policies, but the broader policy “ecosystem”—the constellation of laws, regulations, and sociopolitical norms that together create environments of protection or risk. For instance, rather than analyzing single policies in isolation, research in this collection emphasizes how the sum total of state-level political climates,^4,5^ county-level social spending,^6^ or the aggregation of protections for vulnerable populations like LGBTQ individuals,^7,8^ reverberate through health behaviors and outcomes. While such research is more difficult to translate into policy implications for decision makers because it does not identify the impacts of a single policy, it nevertheless informs theoretical frameworks by recognizing that the environment in which people live is the product of intersecting policies whose interactions often matter more than any single program.

Equally important is the move to critically examine consistency and equity in policy implementation. Traditional evaluation often assumes policies, once enacted, are experienced uniformly across populations. Yet, as discussed in this collection, differential enforcement—by race, geography, or social status—can obscure or even invert apparent effects.^9^ This builds on and extends previous discussions about effect heterogeneity, recasting it as a problem of policy definition and context rather than merely a statistical concept. The implications are clear: for research to inform action, it must attend to the ways in which policy intent, implementation, and lived experience diverge, especially among marginalized groups.

## From policy intent to policy impact: barriers, narratives, and mechanisms of inequity

Moving from broad policy environments to on-the-ground realities, another cross-cutting theme is the “implementation gap,” ie, the difference between what policies promise in theory and what they achieve in practice. For example, there are documented shortfalls in take-up of and access to US social safety net programs,^10,11^ and the articles here probe the challenge in greater depth, surfacing new insights into both barriers and facilitators.

Several papers detail the bureaucratic, logistical, and social hurdles that individuals face in accessing benefits and that organizations face in complying with new rules. The collection expands understanding of these barriers from an individual-level phenomenon to a systems-level dynamic, illustrating how implementation processes themselves may exacerbate or alleviate inequities. For example, administrative burdens, such as complex application processes or confusing eligibility rules, adversely affected employers’ implementation of paid leave initiatives.^12^ Meanwhile, systems-level interventions in the form of nurse home visiting programs increased take-up of critical food assistance benefits during the perinatal and postpartum periods.^13^

The collection also highlights the central, often underappreciated, role of narratives—especially media portrayals and public discourse—in shaping the implementation and perceived legitimacy of social policies. News coverage not only reflects but reinforces societal attitudes about policies like Supplemental Nutrition Assistance Program (SNAP) restrictions for individuals with past felony convictions,^14^ potentially contributing to stigmatization, political will, and ultimately, the design and reach of future interventions.

Taken together, these studies shift the field from a narrow focus on policy evaluation toward a broader analytic lens that includes implementation, administrative design, social context, and narratives. The result is a richer, more actionable picture of how policies succeed or fall short in shaping health and reducing inequities, and how policies are translated from law to lived experience.

## Future directions: charting a course for impactful policy research

As the field evolves, the insights from this collection point toward five key priorities for future social policy and health research:

1. Nuanced Measurement of Policy Contexts and Exposures

Evaluating policy demands approaches that move beyond binary or single-policy indicators to capture complex, dynamic policy environments, variation in implementation, and cumulative exposures over time and space.

2. Integration of Equity and Subgroup Analysis

Research must purposefully examine not just average effects but also differential impacts across racial, socioeconomic, gender, and other axes of identity and geography—disentangling whether observed inequities are due to unequal exposure, enforcement, or underlying vulnerability.

3. Focus on Implementation Science and Administrative Burden

Understanding the “last mile” of policy—including how policies are communicated, administered, and accessed—remains indispensable. Mixed methods, including qualitative inquiry and community partnerships, are needed to illuminate where and why implementation falls short.

4. Recognition of Political and Narrative Determinants

The political context of policy enactment and the narratives that surround social policies are not background noise; they are central drivers of policy scope, sustainability, and effectiveness. Future research should engage political determinants and media discourse as core components of policy analysis.

5. Bridging Silos through Intersectoral Approaches

The complexity of social determinants of health—and their policy levers—demands integrative, cross-sectoral research that incorporates data and perspectives from health, education, criminal justice, social services, and beyond. Such approaches will be vital for designing, evaluating, and scaling interventions with population-level impact.

## Conclusion

The contributions in this special collection propel the literature forward on multiple fronts: clarifying impacts of social policies and broader policy contexts, exposing mechanisms of persistent inequities, and mapping the terrain for future action. Research such as this is particularly important as US federal policy changes result in reduced funding and new administrative burdens for many safety net programs and as media conversations about social policies become increasingly charged. As the boundaries of public health continue to blur with those of politics and social science, research must adapt—becoming at once more rigorous and more attuned to context, experience, and equity.

## Supplementary Material

qxaf193_Supplementary_Data
